# P-901. Clinical Characteristics and Outcomes of CNS Nocardiosis Differ by *Nocardia* Species: A Review of Published Cases

**DOI:** 10.1093/ofid/ofae631.1092

**Published:** 2025-01-29

**Authors:** Michael S Abers

**Affiliations:** National Institutes of Health, Bethesda, Maryland

## Abstract

**Background:**

Nocardiosis is an opportunistic infection with a predilection for central nervous system (CNS) involvement. A large number of *Nocardia* species ( >50) have been associated with human infection. Efforts to compare the clinical characteristics of nocardiosis across *Nocardia* species have been limited by insufficient sample size.

Distribution of Nocardia species/species complex among all patients with CNS nocardiosis.

**Figure:**
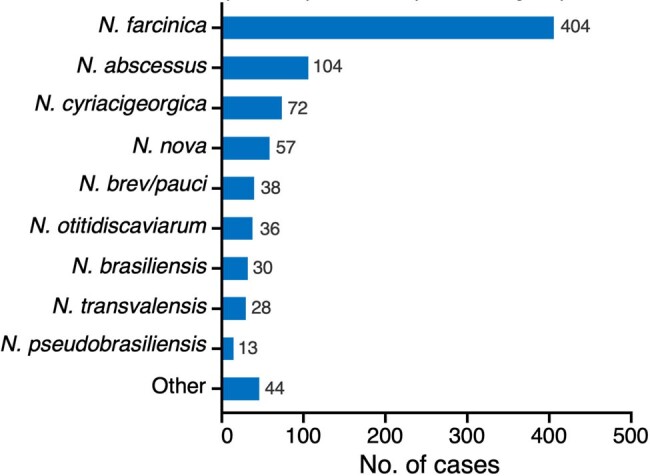

**Methods:**

A literature search of multiple databases (PubMed, Scopus, Google Scholar, Google) was conducted to identify potential cases of CNS nocardiosis published from 1/2000-3/2024. CNS nocardiosis was defined as either identification of *Nocardia* in a CNS specimen or identification of *Nocardia* at any site combined with clinical, laboratory, radiographic, or histopathologic evidence of CNS infection. Cases of CNS nocardiosis were included if *Nocardia* species was reported to at least the species complex level. Duplicate cases were excluded. Categorical variables were compared by chi-squared test with statistical significance defined as P< 0.05.

Frequency of each Nocardia species/species complex stratified by risk factor group.

**Figure:**
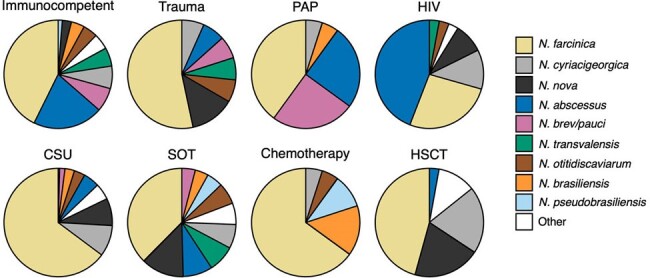

**Results:**

In total, 826 cases of CNS nocardiosis were included; one-third were derived from the grey literature (sources not included in PubMed). Cases were reported in 54 countries with 81.1% from North America, Europe, or East Asia. Half of all cases were caused by *N. farcinica* (**Figure 1**). Brain abscess +/- meningitis was the most common manifestation of CNS nocardiosis (96.4%). The frequency of various characteristics differed across species (**Table 1, Figure 2**). *N. paucivorans/brevicatena* cases were disproportionately reported in Oceania. *N. abscessus* and *N. paucivorans/brevicatena* were overrepresented in immunocompetent individuals and among patients with pulmonary alveolar proteinosis (PAP). These species were associated with a lower mortality rate. *N. nova* was particularly among recipients of solid organ (SOT) or hematopoietic stem cell transplantation (HSCT). Skin and musculoskeletal involvement were more common in patients with *N. brasiliensis*.

Frequency of clinical characteristics for each Nocardia species/species complex.

**Figure:**
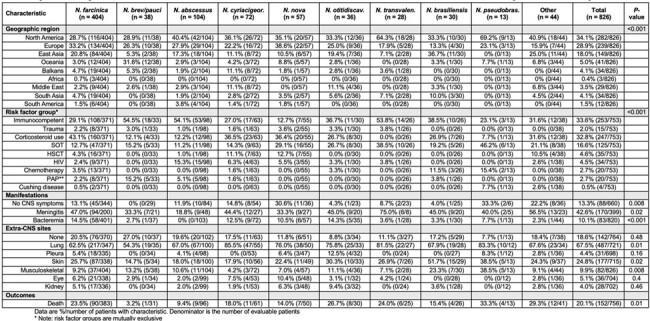

**Conclusion:**

Patient population, clinical manifestations, and outcome of CNS nocardiosis varies according to the species of *Nocardia* responsible for infection. Knowledge of the causative species has important implications for clinical practice.

**Disclosures:**

**All Authors**: No reported disclosures

